# Industrial Processing Induces Pericardial Patch Degeneration

**DOI:** 10.3389/fsurg.2022.881433

**Published:** 2022-05-27

**Authors:** Armin Darius Peivandi, Sven Martens, Barbara Heitplatz, Alena Guseva, Klaus-Michael Mueller, Sabrina Martens

**Affiliations:** ^1^Department of Cardiothoracic Surgery, University Hospital Muenster, Muenster, Germany; ^2^Gerhard-Domagk-Institute of Pathology, University Hospital Muenster, Muenster, Germany

**Keywords:** congenital heart surgery, patches, histopathology, degeneration, industrial processing

## Abstract

**Background:**

Autologous pericardium is considered gold standard for various reconstructive surgical procedures in children. However, processed bovine, equine, and porcine pericardial tissue are also widely used. We investigated structural differences and analyzed alterations caused by industrial processing. Additionally human and equine pericardium explants, used during aortic valve reconstruction were analyzed.

**Methods:**

Pericardial tissues (native, processed and explanted) were gathered and stained with HE and EvG to visualize collagen as well as elastic fibers. Fiber structures were visualized by light and polarization microscopy. Antibody staining against CD 3, CD 20, and CD 68 was performed to identify inflammation.

**Results:**

Native pericardium of different species showed small differences in thickness, with bovine pericardium being the thickest [bovine: 390 μm (± 40.6 μm); porcine: 223 μm (± 30.1 μm); equine: 260 μm (± 28.4 μm)]. Juvenile pericardium was 277 μm (± 26.7 μm). Single collagen bundle diameter displayed variations (~3–20 μm). Parallel collagen fibers were densely packed with small inter-fibrillary space. After industrial tissue processing, loosening of collagen network with inter-fibrillary gapping was observed. Pericardium appeared thicker (mean values ranging from 257–670 μm). Processed tissue showed less birefringence under polarized light. All analyzed tissues showed a small number of elastic fibers. Fibrosis, calcification and inflammatory processes of autologous and equine pericardium were observed in patient explants.

**Conclusion:**

None of the analyzed tissues resembled the exact structure of the autologous pericardial explant. Degeneration of pericardium starts during industrial processing, suggesting a potential harm on graft longevity in children. A careful surgical approach prior to the implantation of xenografts is therefore needed.

## Introduction

In reconstructive surgery for congenital heart disease, different types of pericardial tissues are frequently used. When available, autologous pericardium remains the tissue of choice for multiple indications. However, as many patients need more than one operation, alternative materials are urgently needed. In recent decades, miscellaneous materials and patches have been launched. Many pros and cons have already been discussed. A systematic histopathological analysis is still lacking.

The following commonly used patches are made of either equine, bovine or porcine pericardium:

Matrix patch™ (Auto Tissue GmbH Berlin, Berlin, Germany) is a decellularized equine pericardial patch, with a low RNA and DNA content, fixed in glutaraldehyde (1). CardioCel^®^ (LeMaitre Vascular, Inc., Burlington, MA, USA), a bovine pericardial patch, is treated with the so-called APAPT^®^ anti-calcification process including removal of cell particles and nucleic acids as well as minimization of glutaraldehyde cytotoxicity levels (2). Manufacturers of Supple Peri-Guard^®^ and Vascu-Guard^®^ Patches (LaMed, Oberhaching, Germany), other patches of bovine origin, put an emphasis on the “acellular” aspect of the patch and its permanent tear strength (3). Glutaraldehyde treatment is used for processing of BioIntegral Curved No-React® porcine patch (4). For most industrial patches information on the production process is only available on the companies' websites (5–7).

In this study, we investigated native pericardial tissue of different species. We then compared the histological structure of the native tissue to the industrially processed products. Thus, we analyzed how industrial processing alters pericardium even before implantation.

Secondly, patient explants, gathered from aortic valve re-operations, were examined to investigate further degenerative processes of autologous and equine pericardium after implantation.

## Materials and Methods

### Native and Industrially Processed Material

Native animal pericardium was obtained from a local butcher and histologically compared to industrially processed patch material. Native human pericardium was obtained from a 6-month-old-child during surgery.

### Patient Explants

Patient explants were gathered from 2015 to 2019. All 10 patients had previously undergone aortic valve leaflet reconstruction via patch repair. Written informed consent was obtained directly from adult patients or from parents of minors.

### Ethical Approval

Ethical approval was granted by the local ethics committee (Ethik-Kommission der Ärztekammer Westfalen-Lippe und der Westfälischen Wilhelms-Universität, 2015-231-f-S).

### Methods

Our study consisted of macroscopic and microscopic analyses. Histologic preparation and staining were conducted according to standard procedures as previously reported (8). Staining included Hematoxylin and Eosin (H&E Dye Kit, Art. No. 12156) and Elastica van Gieson (EvG Pikrofuchsin Dye Kit, Art. No. 12739), from MORPHISTO Evolutionsforschung und Anwendung GmbH, Frankfurt am Main, Germany.

Inflammatory processes were identified via immunohistochemistry, using the following antibodies: anti-CD 3 (monoclonal, mouse anti-human, Lot F001, OriGene Technologies, Inc., Rockville, MD, USA), anti-CD 20 (monoclonal, mouse anti-human, baboon, monkey, Lot No. 931-4CS201230, antibodies-online GmbH, Aachen, Germany), anti-CD 68 (monoclonal, mouse anti-human, Lot No. 130 902, antibodies-online GmbH, Aachen, Germany).

BX-43 (Olympus Corporation, Shinjuku, Tokyo, Japan) was used for light and polarization microscopy.

### Statistics

The study presented is of retrospective observational design. Histo(-patho-)logy of different materials currently in use in cardiac surgery was examined.

All specimen were analyzed systematically: Multiple slides of each species were examined and 3 different random locations in each slide were chosen for measurement. Thickness was measured in μm.

Data in the main text is presented as mean (± standard deviation) or median (IQR quartile 3-1). Normality of continuous variables was assessed using Shapiro-Wilk-Test.

## Results

### Native Pericardium of Different Species

**Figure 1:**
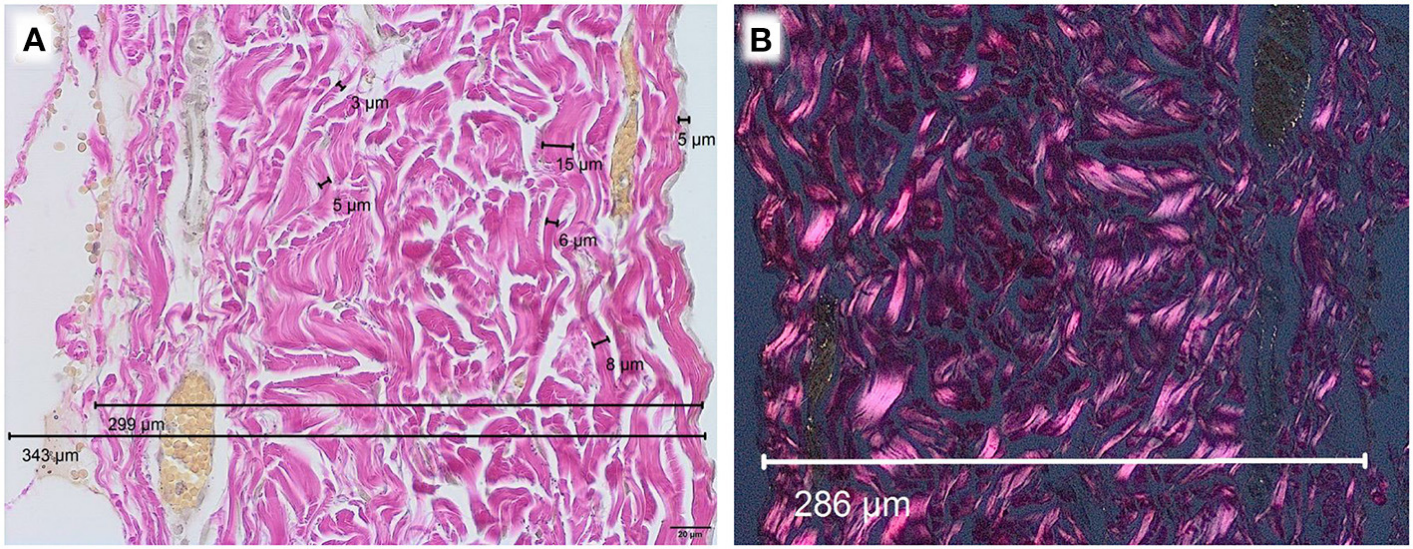
Human pericardium of a 6-month old child, EvG staining, Obj. 40x, **(A)** light microscopy, **(B)** polarization microscopy.

**Figure 2:**
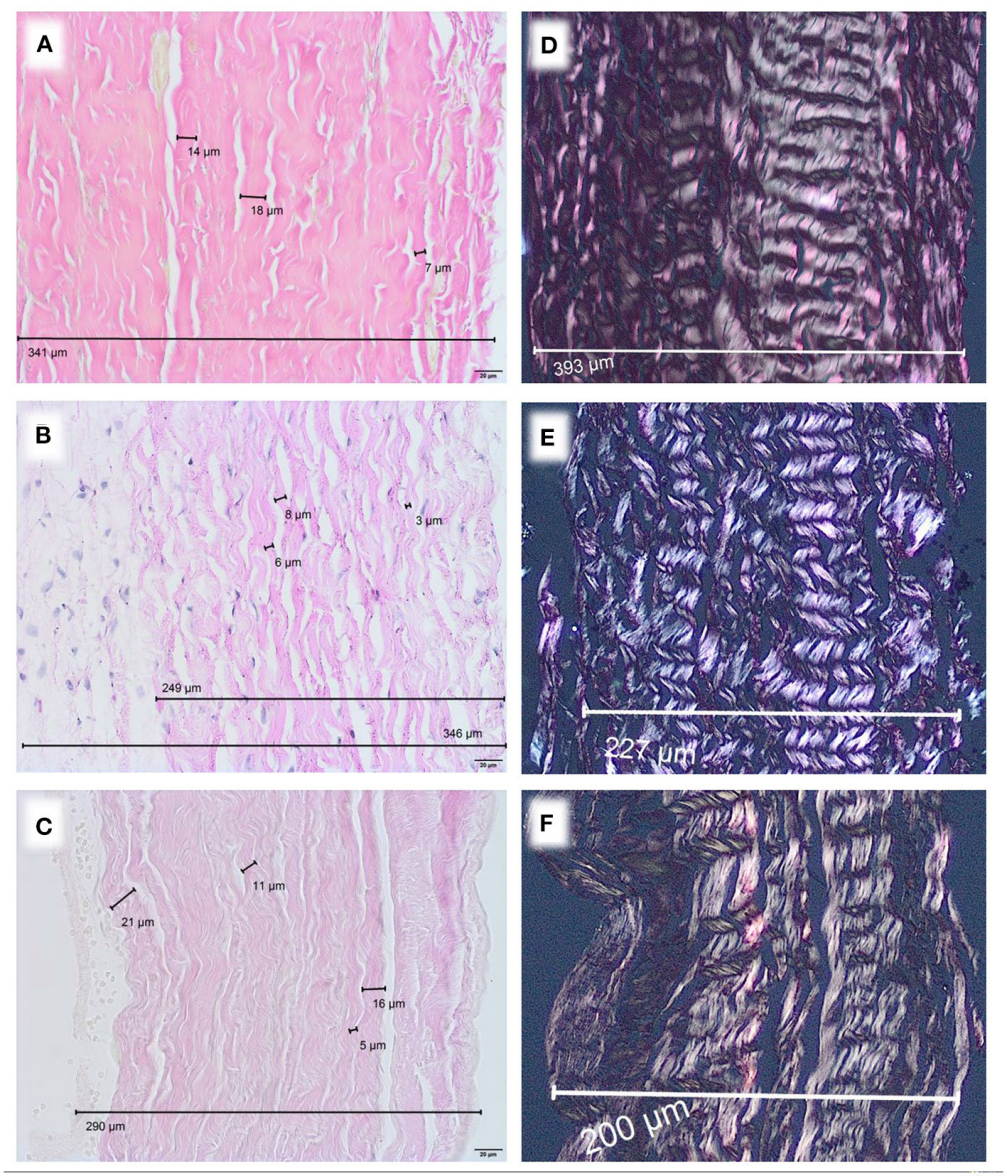
Native pericardium of different species, EvG staining, Obj. 40x, **(A)** bovine, **(B)** porcine, **(C)** equine. Polarization, EvG staining, Obj. 20x, **(D)** bovine, **(E)** porcine, **(F)** equine.

Microscopically, a variation of diameters was observed. Human pericardium of a 6-month-old child showed a mean diameter of 277 μm (± 26.7 μm) (Figure 1A). Bovine pericardium was thickest at 390 μm (± 40.6 μm) (Figure 2A). Porcine and equine pericardium showed a similar thickness [porcine: 223 μm (± 30.1 μm); equine: 260 μm (± 28.4 μm)] (Figures 2B,C).

The single collagen bundle diameters in all different species displayed small variations between approx. 3–20 μm. The parallel collagen fibers were densely packed with only small inter-fibrillary space. All analyzed tissues showed only a very small number of elastic fibers.

Birefringence under polarized light was present in all native specimen (Figures 1B, 2D–F). This can be seen as an indicator for an intact collagen fiber network.

### Industrially Processed Pericardial Patches

**Figure 3:**
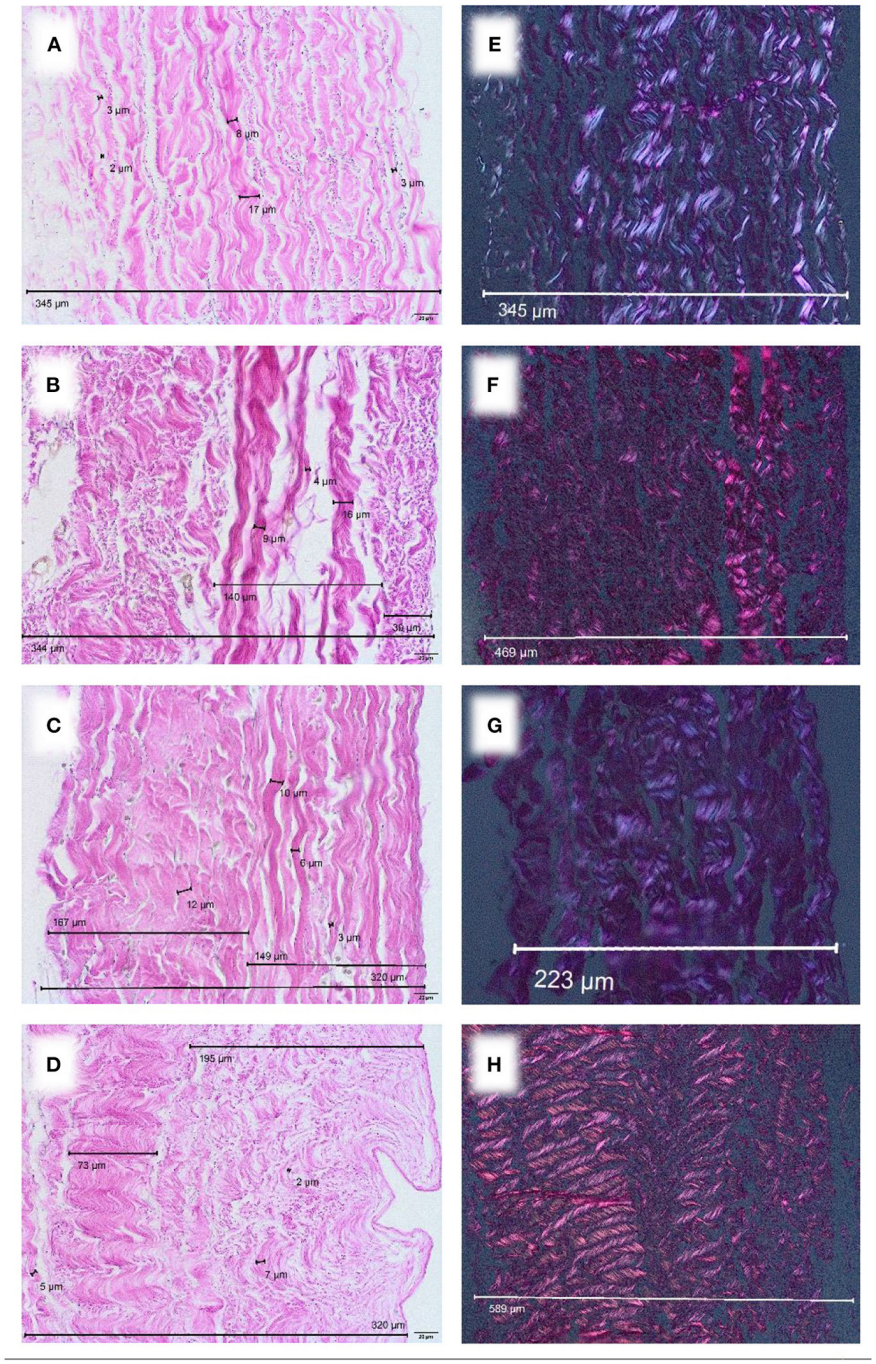
Commercially available patches of different species, EvG staining, Obj. 40x. **(A)** CardioCel^®^ patch (bovine), **(B)** VascuGuard^®^ patch (bovine), **(C)** BioIntegral Curved No-React® patch (porcine), **(D)** Matrix patch™ (equine). Polarization, EvG staining, Obj. 20x. **(E)** CardioCel^®^ patch (bovine), **(F)** VascuGuard® patch (bovine), **(G)** BioIntegral Curved No-React^®^ patch (porcine), **(H)** Matrix patch™ (equine).

After industrial tissue processing, a loosening of the collagen network with inter-fibrillary gapping was observed. This gave processed pericardium a thicker appearance (Figures 3A–D). While processed bovine pericardium ranged from 257–500 μm [CardioCel® 328 μm (± 50.3 μm); Vascu-Guard^®^ 463 μm (± 27.3 μm)], processed porcine pericardium (BioIntegral Curved No-React® porcine patch) displayed a mean thickness of 269 μm (± 30.6 μm). The thickness of equine pericardium (Matrix Patch^TM^) more than doubled after processing [582 μm (± 54.4 μm)].

*Processed* tissue showed less birefringence under polarized light (Figures 3E–H) compared with *native* material (Figures 2D–F) indicating degeneration of the collagen fiber network.

### Degenerative Developments in Patient Explants

After a median duration of implantation of 4.53 (7.42–3.25) years, 10 patients developed stenosis (*n* = 1), insufficiency (*n* = 6) or combined vitia (*n* = 3) of the previously reconstructed aortic valve. In total 8 out of 10 had undergone reconstructive surgery with autologous pericardium. Two other valves were reconstructed with equine pericardium. Detailed clinical patient characteristics are depicted in Table 1.

**Table 1:** Patient characteristics.

**Gender, *n* (%)**	
Female Male	1 (10) 9 (90)
Age at patch implantation (years), median (IQR)	7.92 (12.22–2.98)
Age at reoperation (years), median (IQR)	13.57 (18.82–10.82)
**Primary diagnosis, *n* (%)**	
Combined aortic vitium	3 (30)
Aortic stenosis	7 (70)
**Primary operation, *n* (%)**	
Commissurotomy Balloon valvuloplasty Aortic valve reconstruction	2 (20) 5 (50) 3 (30)
**Secondary operation, *n* (%)**	
Aortic valve reconstruction	7 (70)
**Re-operation, *n* (%)**	
Aortic valve replacement Re-aortic valve reconstruction	8 (80) 2 (20)
**Indication for reoperation**	
Combined aortic vitium With leading insufficiency With leading stenosis Aortic insufficiency	4 (40) 1 (10) 3 (30) 6 (60)
Duration of implantation (years), median (IQR)	4.53 (7.42–3.25)

*IQR, interquartile range*.

Observed patterns of pericardial degeneration included: multi-layer, cushion-like and nodose thickening, (vortex-like) fibrosis, collagen and scattered elastic fibers as well as calcified foci. Substantial neovascularization was not present.

**Figure 4:**
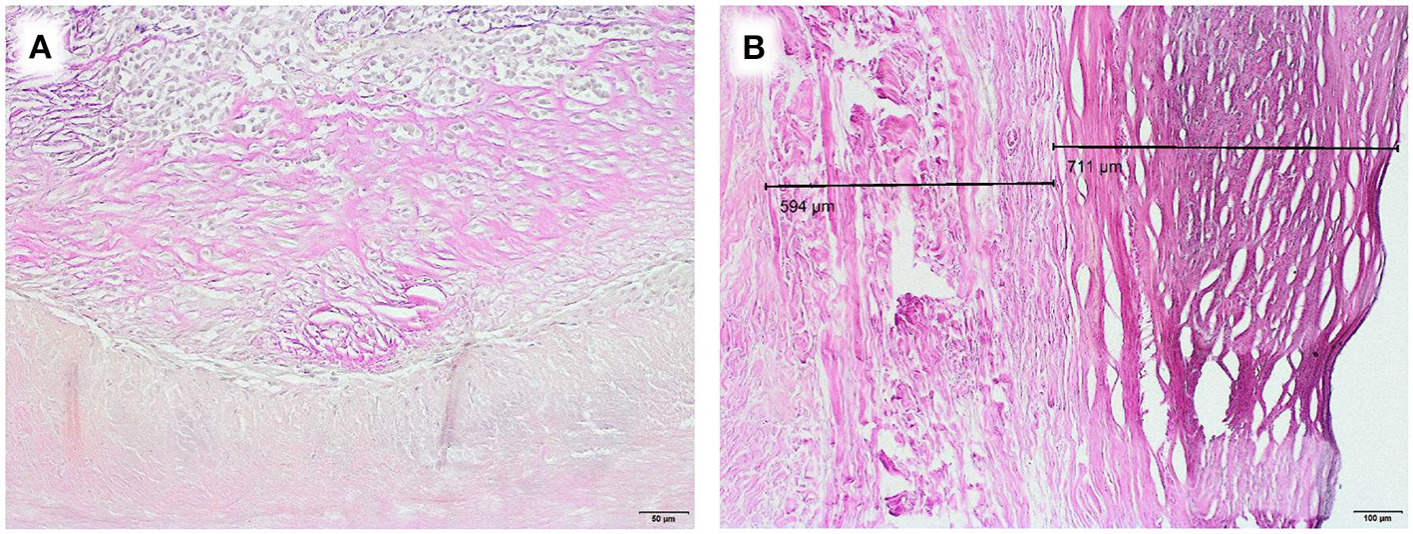
Examples of degenerative developments, EvG staining**, (A)** Chronic foreign body reaction at corporation zone in an equine patch, duration of implantation 5.2 years (Obj. 20x), **(B)** Fibrotic appositions on homologous pericardium, duration of implantation 8.2 years (Obj. 10x).

As an example for systematic degeneration we emphasize two special findings: one equine patch attracted attention with a chronic foreign body reaction at the incorporation zone (Figure 4A). An autologous pericardium explant showed a distinct demarcation line toward fibrotic appositions (Figure 4B).

**Figure 5:**
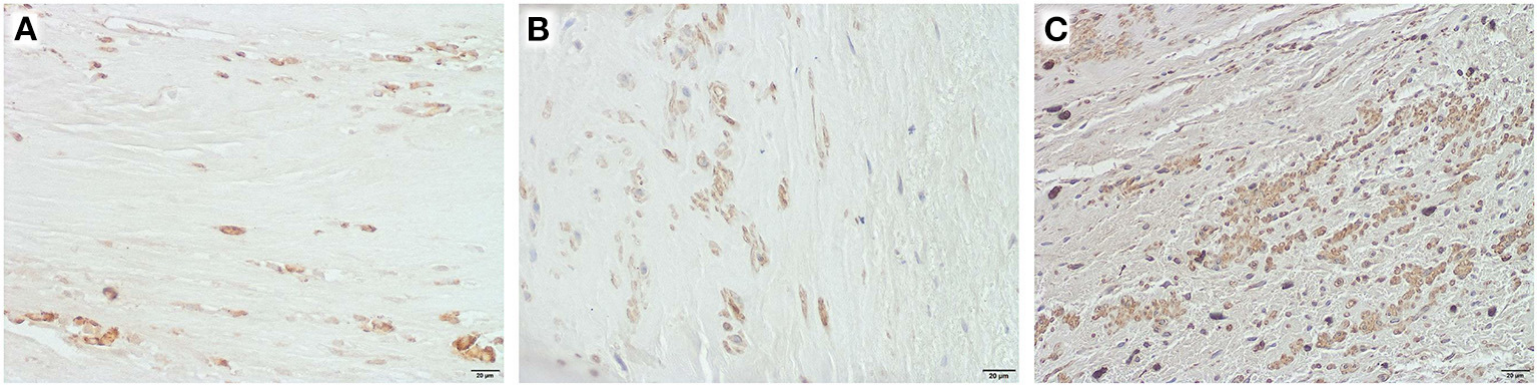
Inflammatory cells in selected explants, Obj. 40x, **(A)** CD 3 positive cells in an equine patch, duration of implantation 5.2 years, **(B)** CD 20 positive cells in autologous pericardium, duration of implantation 4 years, **(C)** CD 68 positive cells in autologous pericardium, duration of implantation 8.2 years.

In half of the specimen, inflammatory cells could be detected (Figure 5). However, the role of these cells in patch degeneration remains unclear, as preoperative serum levels of leukocytes and C-reactive protein were within the normal range in this sub-group [median leukocytes 5.7^*^10^3^/μl (6.25–4.5), median CRP 0.03 mg/dl (0.065–0.02)].

## Discussion

Research on the ideal material for reconstructive surgery in children with congenital heart disease has mainly been focused on clinical outcomes and the frequency of re-operations. Therefore, we wanted to focus on a histologic analysis of different patches to provide a more complete picture of available materials.

The most commonly used industrial patches are of bovine origin, when autologous pericardium is not suitable or available. In recent years, alternative materials were launched to the market. Both porcine (4) and equine (9) patches became increasingly popular among cardiac surgeons. In general, the use of biological material is considered superior to synthetics in terms of biocompatibility and compliance (10).

Our results show that degenerative developments are initiated by processing and storage of pericardium. After implantation in childhood these alterations continue and even aggravate.

In case of bovine pericardium, Nordmeyer et al. reported calcifications in the majority of explanted CardioCel® patches. In addition to that, they discovered pseudo-intima formation, fibroblast ingrowth, occasional inflammatory processes and endothelialization (11). Others discovered fragmentations of collagen matrix, nodular disruption and sclerosis and confirmed calcification of CardioCel®. While they could identify neointima formation, endothelialization of the graft could not be confirmed (2).

Our pre-implant analysis of CardioCel® shows collagen network destruction and tissue loosening. Therefore we hypothesize that ADAPT-treatment provides the basis for degenerative alterations in bovine pericardium. However, other techniques of industrial bovine pericardial processing also showed similar tissue alterations in our analysis (products of Lamed GmbH). Once implanted, they can lead to calcification and inflammation (12).

Clinically, these observations are even more apparent: in a retrospective analysis of the CardioCel® patch, longevity of the implanted material was limited, as freedom from reoperation was only 57% after 3 years (13). In another study, 9 of 60 adult men who received a CardioCel® patches in a valvular position needed reoperation, 6 of them due to focal calcification and consecutive valve dysfunction (2). Pavy et al. experienced graft failure of CardioCel® when implanted in aortic position (14).

We show that processing of porcine pericardium (BioIntegral Curved No-React® porcine patch) also leads to degeneration when compared to native tissue. To our knowledge, there is no systematic histological analysis of this kind of explanted porcine pericardial patch. In children, it is mainly used for aortic reconstruction during Norwood procedure and shows promising early results (4, 15).

In an animal study, Dohmen et al. implanted 7 decellularized equine pericardial scaffolds in juvenile sheep. Four months later, no structural deterioration or degeneration could be observed. The researchers observed a monolayer of endothelial cells on the inner surface of the patches (1).

These positive short term results contradict our observations from explants. We analyzed two equine patches, previously used for aortic valve reconstruction in early childhood. A chronic foreign body reaction at the incorporation zone was present in one sample (Figure 4A). Furthermore, fiber thickening and calcification were detected. In addition to degeneration of the collagen fiber network, the thickness of equine pericardium (Matrix Patch^TM^) already triples after industrial processing (Figures 2C,F, 3D,H).

We chose patients after aortic valve (AoV) repair as this cohort undergoes a rather temporary surgical solution. In general, degeneration, shrinkage and/or calcification limited the longevity of the analyzed patch materials. Nevertheless, with AoV repair during early childhood definite surgical procedures can be postponed. Vergnat et al. reported on 193 patients who needed aortic valve repair due to either stenosis, regurgitation or a combination of both. The surgeons preferred autologous pericardium that was intraoperatively treated with glutaraldehyde 0.3175% for 20 min. The reported results were promising as freedom from reoperation was 89, 70 and 57%, respectively, after 1, 5 and 7 years. The authors hypothesized that the junction of tissues (native and foreign patch material) seen after leaflet extension is more prone to calcification than complete renewed leaflets as stress promotes degeneration (16).

This clinically observed pathomechanism cannot be considered the only explanation for graft failure. In our explants, we encountered a variety of degenerative developments, such as fibrotic appositions (Figure 4B) and calcification.

Our research clearly shows that degeneration starts before implantation. Birefringence as a marker for an intact collagen fiber network is lost during industrial processing. Tissue loosening and inter-fibrillary gapping are observed in all xenografts. None of the analyzed tissues resembled the exact structure of autologous pericardium. A careful surgical approach prior to the implantation of any pericardial patch is therefore needed. Choice of material is not just a question of comfort during surgery. Surgeons should be aware of the limited longevity of all available patches.

## Limitations of This Study

This study compares native pericardium with processed, industrially available patches. As the native pericardium was obtained from a local butcher, the animal breed may not be identical to the one used for industrial purpose by the companies. Furthermore, the explant analysis of this study is only limited to re-operations after aortic valve repair. As our explant analysis was conducted on a small cohort of patients, additional research on this topic is advisable.

Patches at other locations in the heart, i.e. the right ventricular outflow tract, may show different forms of degeneration. It was furthermore our intention to focus on degenerative developments and not on the clinical performance of the analyzed explants. To assess the clinical performance of different patch materials, prospective randomized double blind trials are advisable.

## Data Availability Statement

The raw data supporting the conclusions of this article will be made available by the authors, without undue reservation.

## Ethics Statement

The studies involving human participants were reviewed and approved by Ethik-Kommission der Ärztekammer Westfalen-Lippe und der Westfälischen Wilhelms-Universität, 2015-231-f-S. Written informed consent to participate in this study was provided by the participants' legal guardian/next of kin. The animal study was reviewed and approved by Ethik-Kommission der Ärztekammer Westfalen-Lippe und der Westfälischen Wilhelms-Universität, 2015-231-f-S.

## Author Contributions

AP: writing original draft, data acquisition, formal analysis, and conceptualization. SvM: critical review and editing. BH and K-MM: formal analysis and critical review and editing. AG: data acquisition. SaM: conceptualization, writing original draft, and formal analysis. All authors contributed to the article and approved the submitted version.

## Conflict of Interest

The authors declare that the research was conducted in the absence of any commercial or financial relationships that could be construed as a potential conflict of interest.

## Publisher's Note

All claims expressed in this article are solely those of the authors and do not necessarily represent those of their affiliated organizations, or those of the publisher, the editors and the reviewers. Any product that may be evaluated in this article, or claim that may be made by its manufacturer, is not guaranteed or endorsed by the publisher.
